# Bioremoval of Yttrium (III), Cerium (III), Europium (III), and Terbium (III) from Single and Quaternary Aqueous Solutions Using the Extremophile *Galdieria sulphuraria* (Galdieriaceae, Rhodophyta)

**DOI:** 10.3390/plants11101376

**Published:** 2022-05-22

**Authors:** Manuela Iovinella, Francesco Lombardo, Claudia Ciniglia, Maria Palmieri, Maria Rosa di Cicco, Marco Trifuoggi, Marco Race, Carla Manfredi, Carmine Lubritto, Massimiliano Fabbricino, Mario De Stefano, Seth J. Davis

**Affiliations:** 1 Department of Environmental, Biological and Pharmaceutical Sciences and Technologies, University of Caserta “L.Vanvitelli”, Via Vivaldi 43, 81100 Caserta, Italy; claudia.ciniglia@unicampania.it (C.C.); maria.palmieri@unicampania.it (M.P.); mariarosa.dicicco@unicampania.it (M.R.d.C.); carmine.lubritto@unicampania.it (C.L.); mario.destefano@unicampania.it (M.D.S.); 2 Department of Biology, University of York, Wentworth Way, York YO10 5DD, UK; seth.davis@york.ac.uk; 3 Department of Chemical Sciences, University of Naples “Federico II”, Via Cintia 21, 80126 Naples, Italy; francesco.lombardo@unina.it (F.L.); marco.trifuoggi@unina.it (M.T.); carla.manfredi@unina.it (C.M.); 4 Department of Civil and Mechanical Engineering, University of Cassino and Southern Lazio, Via di Biasio 43, 03043 Cassino, Italy; marco.race@unicas.it; 5 Department of Civil, Architectural and Environmental Engineering, University of Naples Federico II, Via Claudio 21, 80125 Naples, Italy; massimiliano.fabbricino@unina.it; 6 State Key Laboratory of Crop Stress Biology, School of Life Sciences, Henan University, Kaifeng 475004, China

**Keywords:** recycling, rare earth elements, extremophile, *G. sulphuraria*, bioremoval, pH

## Abstract

The lanthanides are among the rare earth elements (REEs), which are indispensable constituents of modern technologies and are often challenging to acquire from natural resources. The demand for REEs is so high that there is a clear need to develop efficient and environmentally-friendly recycling methods. In the present study, living cells of the extremophile *Galdieria sulphuraria* were used to remove four REEs, Yttrium, Cerium, Europium, and Terbium, from single- and quaternary-metal aqueous solutions. Two different strains, SAG 107.79 and ACUF 427, were exposed to solutions buffered at pH 2.5, 3.5, 4.5, and 5.5. Our data demonstrated that the removal performances were strain and pH dependent for all metal ions. At lower pH, ACUF 427 outperformed SAG 107.79 considerably. By increasing the pH of the solutions, there was a significant surge in the aqueous removal performance of both strains. The same trend was highlighted using quaternary-metal solutions, even if the quantities of metal removed were significantly lower. The present study provided the first insight into the comparative removal capacity of the *Galdieria sulphuraria* strains. The choice of the appropriate operational conditions such as the pH of the metal solutions is an essential step in developing efficient, rapid, and straightforward biological methods for recycling REEs.

## 1. Introduction

The successful application of metals in a great variety of fields such as machinery, energy, transportation, building and construction relies on their characteristic features such as high robustness, thermal and electrical conductivity, and great performance at high temperatures [1]. Metals can be repeatedly recycled, decreasing the necessity to extract them from mines [1]. According to the report of the Working Group on the Global Metal Flows to UNEP’s International Resource Panel, the recycling rates of “base metals” (iron, copper, zinc, etc.) are above 50%. In contrast, a large number of metals used in small amounts in new technologies such as red phosphorus, permanent magnets, solar cells, and computer chips are rarely (<1%) recycled [1,2]. At the same time, however, the high popularity of these machineries is causing the demand and price increment of their components, especially of the irreplaceable rare earth elements, REEs [3].

REEs, which comprise 17 metallic elements (15 lanthanides, plus Scandium and Yttrium) with the same chemical properties, have been classified as “critical raw materials” by the European Commission because of their high-supply risk [4]. In many cases, obsolete electrical and electronic equipment can be re-used for advanced technological applications. In these cases, the equipment can be resold or donated to schools or charities without further modifications; alternatively, in particular computers, they can be regenerated—or disassembled into different components, cleaned, repaired and reassembled—and put back on the market, in order to prolong their “life cycle” and reduce the amount of WEEE. When this is not possible, the recycling and recovery of WEEE components have become an essential process to reduce the costs of the disposal and production of the equipment and to minimize the environmental and health risks connected to them.

Physicochemical methods are often used for the recycling and recovery of REEs [5,6], even if some weaknesses exist with these techniques: (1) they are ineffective when the metal concentration is deficient; (2) a large amount of toxic waste will be produced, requiring further treatments, which increases the operating costs and environmental responsibility; (3) some physicochemical methods are costly such as ion exchange and membrane technologies; and (4) incomplete metal adsorption [7]. New methods are thus warranted.

Biosorption and bioaccumulation are biological approaches emerging as promising methods in replacing the physicochemical ones, being eco-friendly and cheaper methods [8]. Biosorption is a metabolism-independent mechanism involving chemical or physical interactions between the metal ions and biosorbent. Despite the high selectivity, metal uptake capacity and reduced sludge production, biosorption is highly dependent on the pH value. This is related to the protonation/deprotonation of the functional groups involved in the binding of the metal ions [9,10]. Bioaccumulation is the intracellular accumulation of metals, and it occurs when cells actively transport them inside the protoplast through an energy (ATP)-driven process [7]. The process is highly economically feasible and selective, even so, the operating conditions (e.g., temperature, pH, and nutrients) need to be strictly controlled to maintain the cell viability [7]. 

Modern technological components and the contaminated effluents used for their production are often made of multiple rare earth elements [11]. Most scientific studies have regarded the bioremoval efficiency in a single-metal system, and little has been examined in a multiple-metal system [12]. The lack of data produces an uncertain estimation of the actual capacity of organisms to recover metals. Mixed-metals effects cannot be predictable from the effects of the single-metals since they depend on extrinsic factors such as the used organism, temperature, pH, metal ion, and biomass concentration [12,13]. In general, a mixture of metals can induce three kinds of behaviour: (1) synergism, when the effect of the mixture is greater than the sum of the individual metal; (2) antagonism, when the effect of the mixture is less than the sum of the individual effects; and (3) lack of interaction when the effect of the mixture is equivalent to the sum of the individual effects [12,14].

Based on these premises, in the present study, two different strains of the extremophile *Galdieria sulphuraria* were tested for the aqueous bioremoval of Yttrium (Y^3+^), Cerium (Ce^3+^), Europium (Eu^3+^), and Terbium (Tb^3+^), important REE constituents of phosphorus lamps, in a single- and quaternary-system. *G. sulphuraria* species are unicellular microalgae strains thriving in geothermal sites, where the ecological conditions are very extreme such as low pH (0.5–3.0), high temperature (50 °C–55 °C), and vast amounts of heavy, precious, and rare earth metals [15,16,17,18]. The coexistence of *G. sulphuraria* and huge amounts of metals in their natural environments makes this microalga one of the best candidates for the biological recovery of metals [19]. Indeed, in the last decade, the interest in using *G. sulphuraria* in the bio-uptake of metals has rapidly grown, thanks to the promising results and the increasingly comprehensive knowledge of their genomes [20,21,22,23]. Data produced in this study primarily aimed to highlight the comparative evaluation of two *G. sulphuraria* strains in terms of the total metal removed in a single-metal system. Quaternary-metal solutions were then used to analyse the influence of the mixed metals on the removal capacity for each metal.

## 2. Results

### 2.1. Removal of Y^3+^, Ce^3+^, Eu^3+^ and Tb^3+^ in the Single-Metal System: The Influence of the Initial pH

In the present paper, the bioremoval of rare metals by two different G. sulphuraria strains was assessed at different initial pH. The pH values were monitored for 24 h both in the control and in the treated samples; a slight decrease (less than 0.5 ± 0.02) was recorded only in the treated samples and remained constant until the end of the experiments (data not shown).

**Figure 1:**
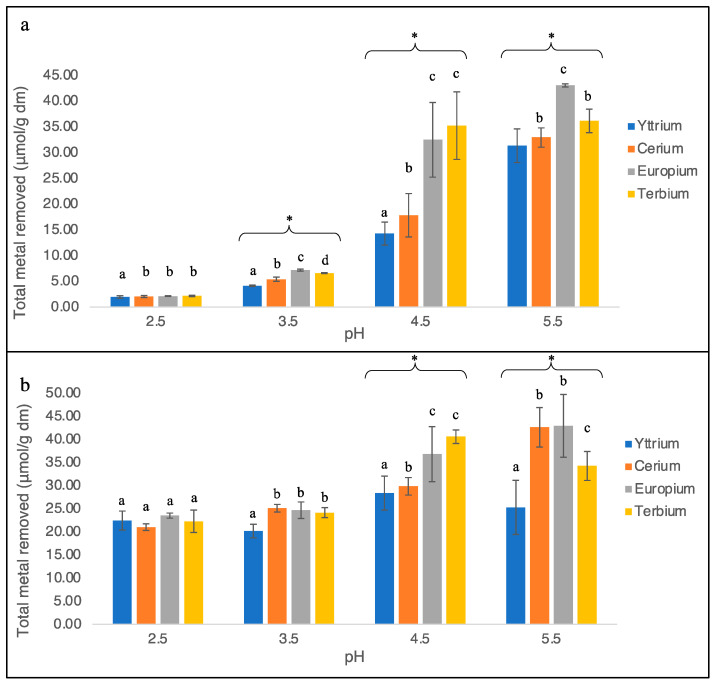
Metal removed from the single-metal aqueous solutions by *G. sulphuraria*, strains SAG 107.79 (**a**) and ACUF 427 (**b**). Data were divided based on the pH of the solutions. Different letters in the same experiment indicate a significant difference, *p* < 0.05; Symbol (*) indicates a *p* < 0.05 significant difference compared to the pH 2.5.

Both *G. sulphuraria* strains SAG 107.79 and ACUF 427 were able to extract the solute metals from the surrounding medium even if the extent of the ability was strain-dependent and metal-dependent. Tests performed in acidic conditions (pH 2.5) highlighted a significant difference in the removal performance amongst the two strains for all of the treatments. While strain SAG 107.79 was able to remove low amounts of each metal (<2.5 µmol/g, Figure 1a), strain ACUF 427 removed 22.43 ± 2.05 µmol/g of Y^3+^, 20.98 ± 0.72 µmol/g of Ce^3+^, 23.49 ± 0.55 µmol/g of Eu^3+^, and 22.26 ± 2.42 µmol/g of Tb^3+^ (Figure 1b). Increasing the medium pH, a gradual rise in the metal removed was observed in SAG 107.79. At a pH of 3.5, 4.13 ± 0.13 µmol/g of Y^3+^, 5.39 ± 0.40 µmol/g of Ce^3+^, 7.18 ± 0.19 µmol/g of Eu^3+^, and 6.60 ± 0.10 µmol/g of Tb^3+^ were removed from the test solutions (Figure 1a). The improved removal performances induced by pH, just described for SAG 107.79, were not highlighted in ACUF 427, which indeed accumulated a comparable metal quantity to those at pH 2.5 (Y^3+^ = 20.13 ± 1.48 µmol/g, Ce^3+^ = 25.08 ± 0.83 µmol/g, Eu^3+^ = 24.66 ± 1.78 µmol/g and Tb^3+^ = 24.14 ± 1.08 µmol/g; Figure 1b). A further increase in pH induced an appreciable boost toward significant quantities of metals being removed for both strains, also showing significant differences among the metals (*p*-value < 0.05). At a pH of 4.5, SAG 107.79 was able to recover 14.26 ± 2.23 µmol/g of Y^3+^, 17.82 ± 4.21 µmol/g of Ce^3+^, 32.45 ± 7.23 µmol/g of Eu^3+^, and 35.21 ± 6.56 µmol/g of Tb^3+^ (Figure 1a), while ACUF 427 removed 28.36 ± 3.68 µmol/g of Y^3+^, 29.82 ± 1.90 µmol/g of Ce^3+^, 36.78 ± 5.95 µmol/g of Eu^3+^, and 40.58 ± 1.47 µmol/g of Tb^3+^ (Figure 1b). The increment of the metals removed from the solutions became even more evident when the tests were performed at the initial pH of 5.5. Indeed, the removed quantities for SAG 107.79 were 31.31 ± 3.28 µmol/g of Y^3+^, 32.91 ± 1.87 µmol/g of Ce^3+^, 43.02 ± 0.32 µmol/g of Eu^3+^, and 36.12 ± 2.26 µmol/g of Tb^3+^ (Figure 1a) and for ACUF 427, they were 25.25 ± 5.87 µmol/g of Y^3+^, 42.60 ± 4.28 µmol/g of Ce^3+^, 42.91 ± 6.80 µmol/g of Eu^3+^, and 34.24 ± 3.13 µmol/g of Tb^3+^ (Figure 1b).

**Figure 2:**
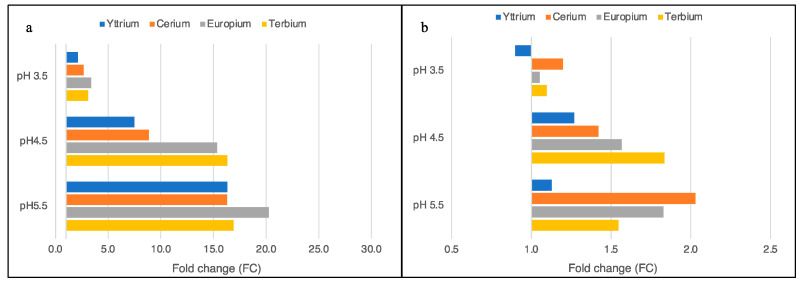
Fold change of the total metal removed from the single-metal aqueous solutions by *G. sulphuraria*, strains SAG 107.79 (**a**) and ACUF 427 (**b**). The fold change was calculated by comparing the μmol/g dm of every metal obtained at pH 3.5, 4.5, and 5.5 with the μmol/g dm measured at pH 2.5.

The influence of the pH on the removal efficiency of each metal species is highlighted in Figure 2a,b. Using SAG 107.79, the Y^3+^ removal increments were a 2.1-fold increase (FC) at pH 3.5, 7.5 FC at pH 4.5, and 16.3 at pH 5.5 (Figure 2a). Similar increments were also obtained with Ce^3+^ (2.7 FC at pH 3.5, 8.9 FC at pH 4.5, and 16.3 at pH 5.5). Higher pH remarkably affected the Eu^3+^ removal from the solutions, as the data demonstrated an increase in the metal removed of 3.4 FC at pH 3.5 and 15.3 FC at pH 4.5 until it reached the increment of 20.3 FC at the highest pH tested (Figure 2a). Similar to the Eu^3+^, the Tb^3+^ removal was hugely affected from pH 4.5, while at pH 5.5, there was no further increase (3.1 FC at pH 3.5, 16.3 FC at pH 4.5, and 16.9 at pH 5.5; Figure 2a).

Unlike SAG 107.79, pH affected the removal efficiency to a lesser extent when using the strain ACUF 427. The Y^3+^ results obtained at pH 3.5 were highly comparable with those at pH 2.5, demonstrating a reduction in the uptake, even if not significant (*p*-value > 0.05). Higher pH induced a slight increase, but this was again not significant (FC < 1.27 for pH 3.5, 4.5, and 5.5; Figure 2b). Smaller increments were observed instead for the other metal species: Ce^3+^ removal showed an FC = 1.20 at pH 3.5, 1.42 at pH 4.5, and 2.03 at pH 5.5 (Figure 2b).

### 2.2. Removal of Y^3+^, Ce^3+^, Eu^3+^ and Tb^3+^ in Quaternary-Metal System: The Combined Effect of the pH and the Simultaneous Presence of the Metals

**Figure 3:**
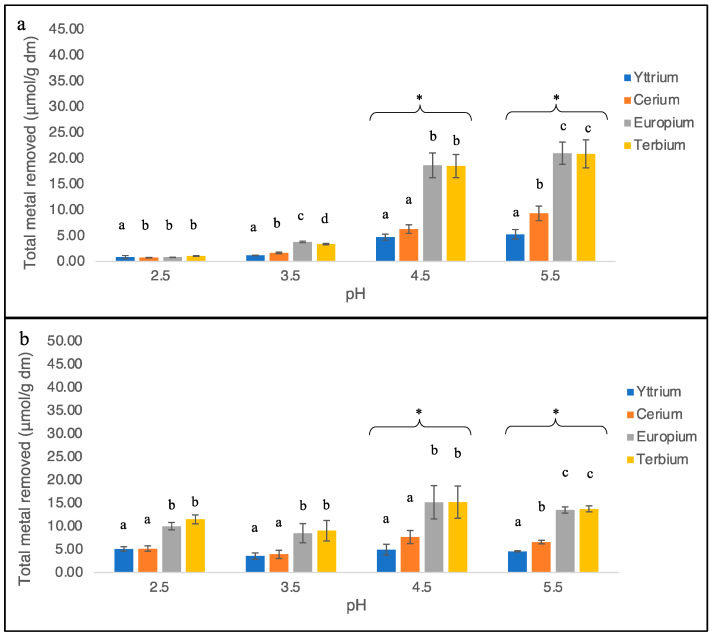
The metal removed from the quaternary-metal aqueous solutions by *G. sulphuraria*, strains SAG 107.79 (**a**) and ACUF 427 (**b**). Data were divided based on the pH of the solutions. Different letters in the same experiment indicate a significant difference, *p* < 0.05; Symbol (*) indicates a *p* < 0.05 significant difference compared to the pH 2.5.

The removal capacity of both strains was also evaluated using quaternary solutions in which the metals were present in equimolar quantities. The total metals removed from the solutions were lower than the single-metal systems, but still, significant differences were highlighted among the metal species and the pH. At pH 2.5, strain SAG 107.79 confirmed the inability to recover metals in appreciable quantities (less than 1.03 µmol/g, Figure 3a), while the ACUF 427 strain reached more significant values (Y^3+^ = 5.08 µmol/g, Ce^3+^ = 5.16 µmol/g, Eu^3+^ = 10.01 µmol/g, Tb^3+^ = 11.47 µmol/g; Figure 3b). Similar to the single solutions, increments in the pH increased the metal bioremoval when SAG 107.79 was used for the assays. Indeed, at pH 3.5, 1.14 ± 0.06 µmol/g of Y^3+^, 1.65 ± 0.15 µmol/g of Ce^3+^, 3.79 ± 0.14 µmol/g of Eu^3+^, and 3.35 ± 0.14 µmol/g of Tb^3+^ were measured in the biomass (Figure 3a). The same increments were not registered for ACUF 427 at the same pH (Y^3+^ = 3.56 ± 0.66 µmol/g, Ce^3+^ = 3.92 ± 0.88 µmol/g, Eu^3+^ = 8.47 ± 2.07 µmol/g, Tb^3+^ = 9.02 ± 2.20 µmol/g; Figure 3b). 

A further increase in the pH at 4.5 deeply influenced the removal rates for both strains as already shown in the single-metal systems, even if to a lesser extent. Metal quantities removed by SAG 107.79 at pH 4.5 were 4.69 ± 0.60 µmol/g of Y^3+^, 6.28 ± 0.84 µmol/g of Ce^3+^, 18.63 ± 2.39 µmol/g of Eu^3+^, and 18.48 ± 2.26 µmol/g of Tb^3+^ (Figure 3a). Comparable quantities were also registered in ACUF 427 (Y3+ = 4.92 ± 1.15 µmol/g, Ce^3+^ = 7.64 ± 1.40 µmol/g, Eu^3+^ = 15.17 ± 3.61 µmol/g, Tb^3+^ = 15.20 ± 3.50 µmol/g; Figure 3b). Finally, at pH 5.5, a slight increase in the metals accumulated was recorded, even if not significantly compared to the previous pH. The total metals removed were 5.26 ± 0.91 µmol/g of Y^3+^, 9.32 ± 1.41 µmol/g of Ce^3+^, 20.98 ± 2.17 µmol/g of Eu^3+^, and 20.85 ± 2.72 µmol/g of Tb^3+^ for SAG 107.79 (Figure 3a) and 4.58 ± 0.12 µmol/g of Y^3+^, 6.59 ± 0.38 µmol/g of Ce^3+^, 13.50 ± 0.68 µmol/g of Eu^3+^, and 13.74 ± 0.64 µmol/g of Tb^3+^ for ACUF 427 (Figure 3b).

**Figure 4:**
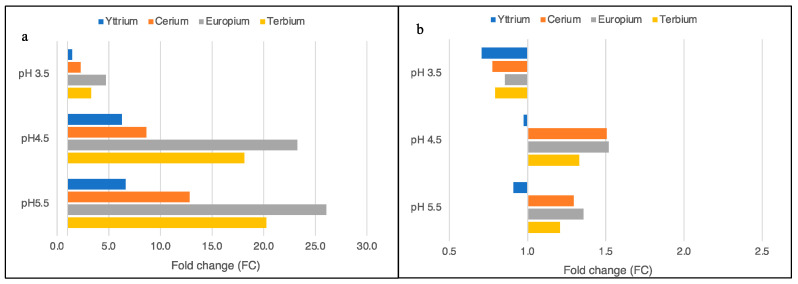
Fold change of the total metal removed from the quaternary-metal aqueous solutions by *G. sulphuraria*, strains SAG 107.79 (**a**) and ACUF 427 (**b**). The fold change was calculated by comparing the μmol/g dm of every metal obtained at pH 3.5, 4.5, and 5.5 with the μmol/g dm measured at pH 2.5.

Using the SAG 107.79 strain, the comparison of the metal quantities at pH 3.5, 4.5, and 5.5 with those at pH 2.5 highlighted a significant increase in the Yttrium removal at pH 4.5 (FC = 6.3), and it remained almost unchanged at pH 5.5 (FC = 6.6). Unlike Yttrium, the ulterior increase in the pH to 5.5 more deeply affected the removal of Ce^3+^, Eu^3+^, and Tb^3+^, even if the rates changed among the metal species (Figure 4a). Indeed, the Cerium quantities increased 2.3 FC at pH 3.5, 8.6 FC at pH 4.5, and 12.8 FC at pH 5.5. Significantly higher quantities were registered for Europium, whose removal rates increased 4.7 times at pH 3.5, 23.3 times at pH 4.5, and up to 26.1 times at pH 5.5 (Figure 4a). As shown for the other metals, the pH solution of 4.5 most influenced the removal of Tb^3+^, increasing the metal quantity of 18.1 FC, while the removed Terbium at pH 5.5 was comparable to that at the previous pH (20.3 FC; Figure 4a). 

A different performance was highlighted when ACUF 427 was used for the assays, demonstrating a weak influence of the pH solution on the metal removal rates. Among the metal species, Yttrium was removed from the microalgae in the same way regardless of the pH (pH 3.5 = 0.7; pH 4.5 = 1.0; pH 5.5 = 0.9; *p*-value > 0.05; Figure 4b). In contrast, the Ce^3+^, Eu^3+^, and Tb^3+^ removal rates were slightly affected by the increase in the pH to 4.5 (Ce^3+^ = 1.5 FC, Eu^3+^ = 1.52 FC, and Tb^3+^ = 1.33 FC). Small increments in the metal quantities were also registered at pH 5.5, reaching an FC of 1.29, 1.36, and 1.21, respectively, for Ce3+, Eu^3+^ and Tb^3+^ (Figure 4b). 

To analyse the effect of the simultaneous presence of the four metals in equimolar quantities on the bioremoval capacity of both strains of *G. sulphuraria*, the total amounts of removed metals were calculated and compared with those obtained with the single-metal solutions. The total metals removed by SAG 107.79 were 148.08 µmol/g at pH 4.5 and 56.41 µmol/g at pH 5.5 (Table S1). In contrast, the total metal quantities calculated in ACUF 427 at different pH did not always exceed those of every single metal. In particular, at pH 2.5, the total amount (31.72 µmol/g) was slightly higher than the single-metal quantities; at pH 3.5, the total amount (24.96 µmol/g) was comparable to the single-metal (Ce^3+^, Eu^3+^ and Tb^3+^; Table S2). At pH 4.5, the total amount of metals removed from the quaternary solution was 42.93 µmol/g, which was statistically higher when compared to the Y^3+^ and Ce^3+^ quantities at the same pH from the single-metal system (Table S2). Finally, at pH 5.5, the total metal removed was 38.41 µmol/g, which did not reach the Eu^3+^ and Ce^3+^ quantities removed from the single-metal system (Table S2).

## 3. Discussion

The ability of algae in recovering REEs from e-waste has been widely examined and assessed in the literature [24]. The removal of REEs is strictly correlated to the physical, chemical, and biological characteristics of the algae. Namely, the algal cell wall is rich in functional groups, which contribute to the high-removal performances, as assessed for different microalgae such as *Desmodesmus multivariabilis*, *Chlorella vulgaris*, and *Chlamydomonas reinhardii* [25] as well as for macroalgae [26,27]. REEs after binding on the cell wall can be transported into cells by carrier proteins and stored in different cell compartments [24].

The utility of microalgae for the biorecycling of REEs from end-of-life products has been predominantly evaluated in a single-metal context. However, in light of a scale-up application of these systems, the main practical interest is the assessment of the microalgal biorecovery capacity in a multi-metal system, which is more likely to be the scenario of industrial effluents [12,28]. In the present study, we investigated the ability of *G. sulphuraria* strains SAG 107.79 and ACUF 427 to accumulate four rare earth metals, Y^3+^, Ce^3+^, Eu^3+^, and Tb^3+^, from aqueous solutions at a wide range of pH (from 2.5 to 5.5) in single- and quaternary-metal systems. We found that the ACUF 427 strain was superior in highly acidic conditions and that both strains performed well under more weakly acidic contexts.

When single-metal solutions were used, significant differences were highlighted between the two strains (*p*-value < 0.05). At pH 2.5, ACUF 427 outperformed SAG 107.79 as it removed metal quantities 10-fold higher than those of the other strain (Figure 1a,b). Higher pH induced a remarkable increase in the metal removal, especially for the SAG 107.79 strain. The fold change, calculated by comparing the quantity of the metals per grams of dried biomass at the chosen pH and pH 2.5, highlighted significant differences among the metal species removed from the solutions. In contrast, increments of the metals removed by ACUF 427, based on the pH solutions, were much lower than those by SAG 107.79, thus hypothesising that the capacity of the metal removal of this strain was less affected by the pH (Figure 2a,b). Few data have been produced in the research on *G. sulphuraria* to study the accumulation of different kinds of metals in single-metal solutions. The *G. sulphuraria* strain ACUF 074 was used for the recovery of a great variety of REEs by varying various parameters such as the concentration of the metal ions, the pH of the experimental solutions, and the preincubation of the microalgal stock solutions [20]. In particular, the authors described a reduction in the REE uptake with increasing pH of the solutions, which is in contrast to the results of the present study. Afterward, they efficiently recovered gold and palladium (over 80%) from aqua regia-based metal wastewater, using 1.4 mg/mL of biomass (dry matter) and an exposition time of 30 min. They also identified a suitable modality for the desorption of metal ions from the biomass using a solution of 1 M thiourea and 0.1 M HCl [21]. *G. sulphuraria* biomass grown under mixotrophic conditions was also able to recover the radionuclide Cesium, reaching a recovery percentage of 52 ± 15%, after 10 days of exposition to the metal [22].

From the quaternary solutions of the metals, a significant reduction in the bioremoval was highlighted for both strains (*p*-value < 0.05), but SAG 107.79 still outperformed ACUF 427 when considering the calculated fold change (Figure 4a). Unlike the results of SAG 107.79, in ACUF 427, the simultaneous presence of four metals at pH 3.5 resulted in a reduction in all the metals, even if not significant. ACUF 427 reached values significantly lower at higher pH than those calculated for SAG 107.79 (Figure 4b). Data from the present study confirmed the results obtained by Čížková et al. (2021), who used *G. phlegrea* to evaluate the simultaneous removal of REEs using luminophores. The concentration of the included metals was not in equimolar quantities, but it precisely reflected the measured metal of the luminophores [29]. Considering the results of every single metal, the authors measured up to 1.98 mg/g, except for Yttrium (11.14 mg/g), which was already present in high concentration in the initial solution of 178.65 mg/g [29].

When the experimental design mainly includes a biosorption approach, a high variety of physicochemical and biological parameters such as metal ionic characteristics (e.g., atomic weight, ion radius, valence, etc.), the nature of the biosorbents (e.g., cell age), and the biosorption conditions (e.g., pH, temperature, contact time, etc.) must be considered. These represent the main reasons for a different removal rate by biomass [30]. Among the metal ion characteristics, the most important are the atomic weight, electronegativity, ionic radius, and covalent index [30,31]. Previous data have demonstrated the positive correlation between the biosorption rates and the atomic weight, being larger ions capable of binding sites with two distant functional groups [31,32]. An increased biosorption was also observed when more electronegative metal ions were used [33]. Different biosorption rates for a high variety of metals were explained by the influence of the covalent index (X^2^_m_r) with the freeze-dried cells of *Rhizopus arrhizus* [34], and *Saccharomyces cerevisiae* [30].

In the present study, a correlation approach was used to understand the influence of the above-mentioned physiochemical properties on the bioremoval degree of *G. sulphuraria*. A positive correlation with the covalent index and the electronegativity was registered at pH 2.5 when the ACUF 427 strain was used (R^2^ = 0.93, *p*-value < 0.05), while no significant correlations were found with the other metal properties and at higher pH (data not shown). Different results were obtained with the SAG 107.79 strain, where only positive correlations were obtained between the metal removal and the ionic radius at a higher pH (R^2^ > 0.91, *p*-value < 0.05; data not shown). The incongruent results obtained from the correlation analysis suggested a more complicated system than the easier biosorption approach. The uptake level also depends on the composition and the specific properties of the cell wall of the microalgae. Important microalgal cell wall components are peptidoglycan, polysaccharides, and proteins [35]. Most of these molecules carry charged groups such as carboxyl, phosphate, hydroxyl, or amine, which could be protonated or deprotonated, depending on the media pH. Oxygen, sulphur, and phosphorus atoms present in these groups perfectly react with rare earth trivalent ions, with the last classified as a Pearson hard acid [27]. This electrostatic attraction plays an essential role in the recovery process and could explain the differential uptake of the two strains of *G. sulphuraria*. 

The recovery of rare earth can also be achieved by bioprecipitation, thanks to the release of organic phosphates, which cause the precipitation of the metal in the form of phosphate (Dev et al., 2020). Moreover, ions could either be attached to the cell surface or transported and accumulated inside the cell, in various contexts [27]. Bioaccumulation is generally enhanced by specific cysteine-rich peptides such as glutathione, metallothioneins, lipopolysaccharides, and phytochelatins [9]. The different removal rates amongst the two strains, especially at pH 2.5, could also be ascribed to the different transportation systems activated by the microalgae. In general, the transport across the membrane is also affected by the physiochemical parameters (e.g., molecular size and polarity) [26,36,37]. Inside the cell, metal ions could accumulate in the vacuoles or be bound by specific molecules for storage or detoxification [27,36,38]. In this regard, the genomic analyses identified in *G. sulphuraria* enzymes such as arsenite methyl transferases and mercury reductase employed in the biotransformation into less toxic and metal derivates [39]; these findings provide a reasonable explanation of the high-metal resistance by this extremophilic alga.

## 4. Materials and Methods

### 4.1. Metal Stock Solutions

In this study, the removal capacity of Ce^3+^, Eu^3+^, Y^3+^, and Tb^3+^ as single- and quaternary-systems was studied using the living algal biomass of *G. sulphuraria* at a constant and equimolar concentration of 178 µmol/L. Y^3+^, Ce^3+^, Eu^3+^, and Tb^3+^ were acquired from Alfa Aesar (USA) in the form of chloride salt monohydrate (MetalCl_3_.H_2_O, 99.9%). Stock solutions were prepared, dissolving 2 g of each metal salt in 1 litre of Milli-Q water and acidified at pH 2.5, 3.5, 4.5, and 5.5 using sulphuric acid (98%). All of the solutions were then sterilised with a 0.45 µm filter. To prevent interferences with the chemical analyses, all materials were previously rinsed with nitric acid and deionised water prior to use. In addition, the initial concentration (C_i_) of the pH adjusted REE solutions were verified by ICP-MS before the experiments to ensure that there was no precipitation involved for the tested REE concentration (178 µmol/L). The pH of the metal solution was measured before and after sterilisation (pHmeter Mettler-Toledo GmbH Process, Switzerland).

### 4.2. Microalgal Culture Preincubation

Two *G. sulphuraria* strains, genetically distant from each other, were used for this study. Strain ACUF 427 was obtained from the algal collection of the University of Naples “Federico II” (www.acuf.net) (accessed on 15 April 2022) and was formerly collected from the acidic soil of the thermal station in Gunnuhver, Southwest Iceland. Strain SAG 107.79, originally sampled from a very hot acidic water in Sonoma, California, was obtained from the Culture Collection of Algae at Göttingen University (https://www.uni-goettingen.de/en/culture+collection+of+algae+%28sag%29/184982.html) (accessed on 15 April 2022). Both algal cultures were further isolated by streaking the colonies three times across Allen agar plates, starting from a diluted solution of the cultures. The ultimate colonies were eluted in Allen medium at pH 2.5 and grown at a temperature of 37 °C and constant light intensity (50 µmol photons/m^2^ s). The cultures were refreshed weekly with a new medium until the microalgae reached the logarithmic growth phase.

### 4.3. Experimental Design

The metal uptake experiments were performed in triplicate, in 24-well plates (Thermo Fisher Scientific, Waltham, MA, USA) 2 mL solutions, stirred on a tilting shaker (MR-1 Biosan, BioScientifica, Rome, Italy), and kept in a climatic chamber (ThermoFisher Scientific, Waltham, MA, USA) at 37 °C (Figure S1). A defined volume of microalgal culture was centrifuged at 13.000 rpm for 2 min at 4 °C; the supernatant was discarded, and the algal pellet was washed twice with autoclaved deionised water and then added to the experimental metal solution (water + metal), in order to achieve an initial optical cell density of 5 OD (ƛ = 550 nm; Secomam spectrophotometer Prim light). Positive controls (metal solutions without microalgal biomass) and negative controls (algal biomass without metals) were also considered. pH of each metal solution was measured at the beginning and at the end of exposure (24 h) (pHmeter Mettler-Toledo GmbH Process, Greifensee, Switzerland).

After 24 h of treatment, the samples were collected and centrifuged at 10,000 rpm to separate the biomass fractions from the supernatant ones. The supernatant samples were filtered with a 0.2 µm filter, while the biomass samples were washed twice with H_2_O at the corresponding pH and digested using aqua regia (HNO_3_:HCl = 3:1 *v/v*). Digestion was conducted in a microwave oven (Milestone OneTouch, Bergamo, Italy) at 175 °C for 10 min, following the U.S. Standard recommendations (US-EPA 3051A). Metal concentrations were finally measured in the supernatant and the digested samples through inductively coupled plasma mass spectrometry using an ICP-MS (Aurora M90 Bruker Daltonics). The evaluation of the metal uptake was conducted by measuring the total metal removed and the removal efficiency. The first measure was carried out using the following formula:

Total metal removed (μmol/g dm) = (C_biomass_ × V/M)/metal molecolar weight

where C_biomass_ is the metal concentration measured in the biomass fraction; V is the volume of the test solutions; and M is microalgal biomass (g, dry matter).

### 4.4. Statistical Analysis

All of the experiments were performed in triplicate and the data were expressed as mean ± standard deviation. Metal recovery values were analysed through one-way analysis of variance (ANOVA). A multiple comparison Tukey test was then used to evaluate the significance of the differences among the treatments.

## 5. Conclusions

Knowing the effect of the pH is one step towards fully understanding the mechanisms involved in the bioremoval of rare earth metals, and the findings of this study represent an added value for developing an efficient system. Based on the data produced and our willingness to speculate on the best conditions in terms of the pH solution and chosen strain of *G. sulphuraria* for each metal and using equimolar quaternary solutions, all four metal species were best removed by SAG 107.79 at pH 5.5. If there is a necessity to use more acidic solutions to remove these metals, the best choice lies in ACUF 427. Further data on the cellular localisation of the metal ions could represent an important step in understanding the contribution of biosorption and bioaccumulation to the bioremoval of REEs using *G. sulphuraria* biomass. 

## Data Availability

The data presented in this study are available on request from the corresponding author. The data are not publicly available due to privacy restrictions.

## Supplementary Materials

The following supporting information can be downloaded at: https://www.mdpi.com/article/10.3390/plants11101376/s1, Figure S1. Example of the triplicate of the treated and untreated samples (green wells) in 24-well plates. The plates were kept on a rotary shaker at 37 °C for 24 h. Transparent-wells contained 2 mL metal solutions set up as the positive control. Table S1. Total metal removed from the single- and quaternary-metal aqueous solutions by the *G. sulphuraria* strain SAG 107.79. Data are expressed as μmol/g dry matter. The total metal removed quantities were calculated by adding the amount of every metal component (Y^3+^ + Ce^3+^ + Eu^3+^ + Tb^3+^). Table S2. The total metal removed from the single- and quaternary-metal aqueous solutions by the *G. sulphuraria* strain ACUF 427. Data are expressed as μmol/g dry matter. The total metal removed quantities were calculated by adding the amount of every metal component (Y^3+^ + Ce^3+^ + Eu^3+^ + Tb^3+^).

## References

[1] Graedel T.E., Allwood J., Birat J.P., Buchert M., Hagelüken C., Reck B.K., Sibley S.F., Sonnemann G. (2011). What do we know about metal recycling rates?. J. Ind. Ecol..

[2] Reck B.K., Graedel T.E. (2012). Challenges in metal recycling. Science.

[3] Binnemans K., Jones P.T., Blanpain B., Van Gerven T., Yang Y., Walton A., Buchert M. (2013). Recycling of rare earths: A critical review. J. Clean. Prod..

[4] European Commission Enterprise and Industry (2010). Critical Raw Materials for the EU—Report of the Ad-hoc Working Group on Defining Critical Raw Materials.

[5] Dhankhar R., Hooda A. (2011). Fungal biosorption-an alternative to meet the challenges of heavy metal pollution in aqueous solutions. Environ. Technol..

[6] Farooq U., Kozinski J.A., Khan M.A., Athar M. (2010). Biosorption of heavy metal ions using wheat based biosorbents—A review of the recent literature. Bioresour. Technol..

[7] Lo Y.C., Cheng C.L., Han Y.L., Chen B.Y., Chang J.S. (2014). Recovery of high-value metals from geothermal sites by biosorption and bioaccumulation. Bioresour. Technol..

[8] Das N. (2010). Recovery of precious metals through biosorption—A review. Hydrometallurgy.

[9] Zeraatkar A.K., Ahmadzadeh H., Talebi A.F., Moheimani N.R., McHenry M.P. (2016). Potential use of algae for heavy metal bioremediation, a critical review. J. Environ. Manag..

[10] Pacheco P.H., Gil R.A., Cerutti S.E., Smichowski P., Martinez L.D. (2011). Biosorption: A new rise for elemental solid phase extraction methods. Talanta.

[11] Menad N., Seron A. (2017). Characteristics of Nd-Fe-B Permanent Magnets Present in Electronic Components. Int. J. Waste Resour..

[12] Monteiro C.M., Castro P.M., Malcata X.F. (2011). Capacity of simultaneous removal of zinc and cadmium from contaminated media, by two microalgae isolated from a polluted site. Environ. Chem. Lett..

[13] Franklin N.M., Stauber J.L., Lim R.P., Petocz P. (2002). Toxicity of metal mixtures to a tropical freshwater alga (Chlorella Sp.): The Effect of interactions between Copper, Cadmium, and Zinc on Metal Cell Binding and Uptake. Environ. Toxicol. Chem..

[14] Qi B.C., Aldrich C. (2008). Biosorption of heavy metals from aqueous solutions with tobacco dust. Bioresour. Technol..

[15] Del Mondo A., Iovinella M., Petriccione M., Nunziata A., Davis S.J., Cioppa D., Ciniglia C. (2019). A Spotlight on Rad52 in Cyanidiophytina (Rhodophyta): A Relic in Algal Heritage. Plants.

[16] Iovinella M., Carbone D.A., Cioppa D., Davis S.J., Innangi M., Esposito S., Ciniglia C. (2020). Prevalent pH controls the capacity of Galdieria maxima to use ammonia and nitrate as a nitrogen source. Plants.

[17] Eren A., Iovinella M., Yoon H.S., Cennamo P., de Stefano M., de Castro O., Ciniglia C. (2018). Genetic structure of Galdieria populations from Iceland. Polar Biol..

[18] Ciniglia C., Yang E.C., Pollio A., Pinto G., Iovinella M., Vitale L., Yoon H.S. (2014). Cyanidiophyceae in Iceland: Plastid rbc L gene elucidates origin and dispersal of extremophilic *Galdieria sulphuraria* and *G. maxima* (Galdieriaceae, Rhodophyta). Phycologia.

[19] di Cicco M.R., Iovinella M., Palmieri M., Lubritto C., Ciniglia C. (2021). Extremophilic Microalgae Galdieria Gen. for Urban Wastewater Treatment: Current State, the Case of “POWER” System, and Future Prospects. Plants.

[20] Minoda A., Sawada H., Suzuki S., Miyashita S.-i., Inagaki K., Yamamoto T., Tsuzuki M. (2015). Recovery of rare earth elements from the sulfothermophilic red alga *Galdieria sulphuraria* using aqueous acid. Appl. Microbiol. Biotechnol..

[21] Ju X., Igarashi K., Miyashita S.-i., Mitsuhashi H., Inagaki K., Fujii S.-I., Sawada H., Kuwabara T., Minoda A. (2016). Effective and selective recovery of gold and palladium ions from metal wastewater using a sulfothermophilic red alga, *Galdieria sulphuraria*. Bioresour. Technol..

[22] Fukuda S.-y., Yamamoto R., Iwamoto K., Minoda A. (2018). Cellular accumulation of cesium in the unicellular red alga Galdieria sulphuraria under mixotrophic conditions. J. Appl. Phycol..

[23] Sirakov M., Palmieri M., Iovinella M., Davis S.J., Petriccione M., di Cicco M.R., De Stefano M., Ciniglia C. (2021). Cyanidiophyceae (Rhodophyta) Tolerance to Precious Metals: Metabolic Response to Palladium and Gold. Plants.

[24] Cao Y., Shao P., Chen Y., Zhou X., Yang L., Shi H., Yu K., Luo X., Luo X. (2021). A critical review of the recovery of rare earth elements from wastewater by algae for resources recycling technologies. Resour. Conserv. Recycl..

[25] Birungi Z.S., Chirwa E.M.N. (2014). The kinetics of uptake and recovery of lanthanum using freshwater algae as biosorbents: Comparative analysis. Bioresour. Technol..

[26] Pinto J., Henriques B., Soares J., Costa M., Dias M., Fabre E., Lopes C.B., Vale C., Pinheiro-Torres J., Pereira E. (2020). A green method based on living macroalgae for the removal of rare-earth elements from contaminated waters. J. Environ. Manag..

[27] Jacinto J., Henriques B., Duarte A.C., Vale C., Pereira E. (2018). Removal and recovery of Critical Rare Elements from contaminated waters by living Gracilaria gracilis. J. Hazard. Mater..

[28] Birungi Z.S., Chirwa E.M.N., Botai O.J. (2017). Competitive adsorption in a ternary system of toxic metals and rare earth elements using Desmodesmus multivariabilis: Empirical and kinetic modelling. J. Appl. Phycol..

[29] Čížková M., Mezricky P., Mezricky D., Rucki M., Zachleder V., Vítová M. (2021). Bioaccumulation of Rare Earth Elements from Waste Luminophores in the Red Algae, *Galdieria phlegrea*. Waste Biomass Valorization.

[30] Chen C., Wang J. (2007). Influence of metal ionic characteristics on their biosorption capacity by *Saccharomyces cerevisiae*. Appl. Microbiol. Biotechnol..

[31] Prasher S.O., Beaugeard M., Hawari J., Bera P., Patel R.M., Kim S.H. (2004). Biosorption of heavy metals by red algae (*Palmaria palmata*). Environ. Technol..

[32] Haug A., Smidsrod O. (1970). Selectivity of Some Anionic Polymers for Divalent Metal Ions. Acta Chem. Scand..

[33] Chong K.H., Volesky B. (1995). Description of two-metal biosorption equilibria by Langmuir-type models. Biotechnol. Bioeng..

[34] Brady J.M., Tobin J.M. (1995). Binding of hard and soft metal ions to *Rhizopus arrhizus* biomass. Enzyme Microb. Technol..

[35] Bailey R.W., Staehelin L.A. (1968). The chemical composition of isolated cell walls of *Cyanidium caldarium*. J. Gen. Microbiol..

[36] Hao S., Xiaorong W., Liansheng W., Lemei D., Zhong L., Yijun C. (1997). Bioconcentration of Rare Earth Elements lanthanum, gadolinium and yttrium in algae (Chlorella Vulgarize Beijerinck): Influence of chemical species. Chemosphere.

[37] Tan Q.G., Yang G., Wilkinson K.J. (2017). Biotic ligand model explains the effects of competition but not complexation for Sm biouptake by *Chlamydomonas reinhardtii*. Chemosphere.

[38] Kumar D., Pandey L.K., Gaur J.P. (2016). Metal sorption by algal biomass: From batch to continuous system. Algal Res..

[39] Schönknecht G., Chen W., Ternes C.M., Barbier G.G., Shrestha R.P., Stanke M., Bräutigam A., Baker B.J., Banfield J.F., Garavito R.M. (2013). Gene Transfer from Bacteria and Archaea Facilitated Evolution of an Extremophilic Eukaryote. Science.

