# Supplementary material for: Bioremoval of Yttrium (III), Cerium (III), Europium (III), and Terbium (III) from Single and Quaternary Aqueous Solutions Using the Extremophile Galdieria sulphuraria (Galdieriaceae, Rhodophyta)
Source: Plants (Basel). 2022 May 22;11(10):1376. doi: 10.3390/plants11101376 (PMC9144214; doi:10.3390/plants11101376)
Supplement: Supplementary file 1 [file plants-11-01376-s001.zip › FigureS1.pdf]

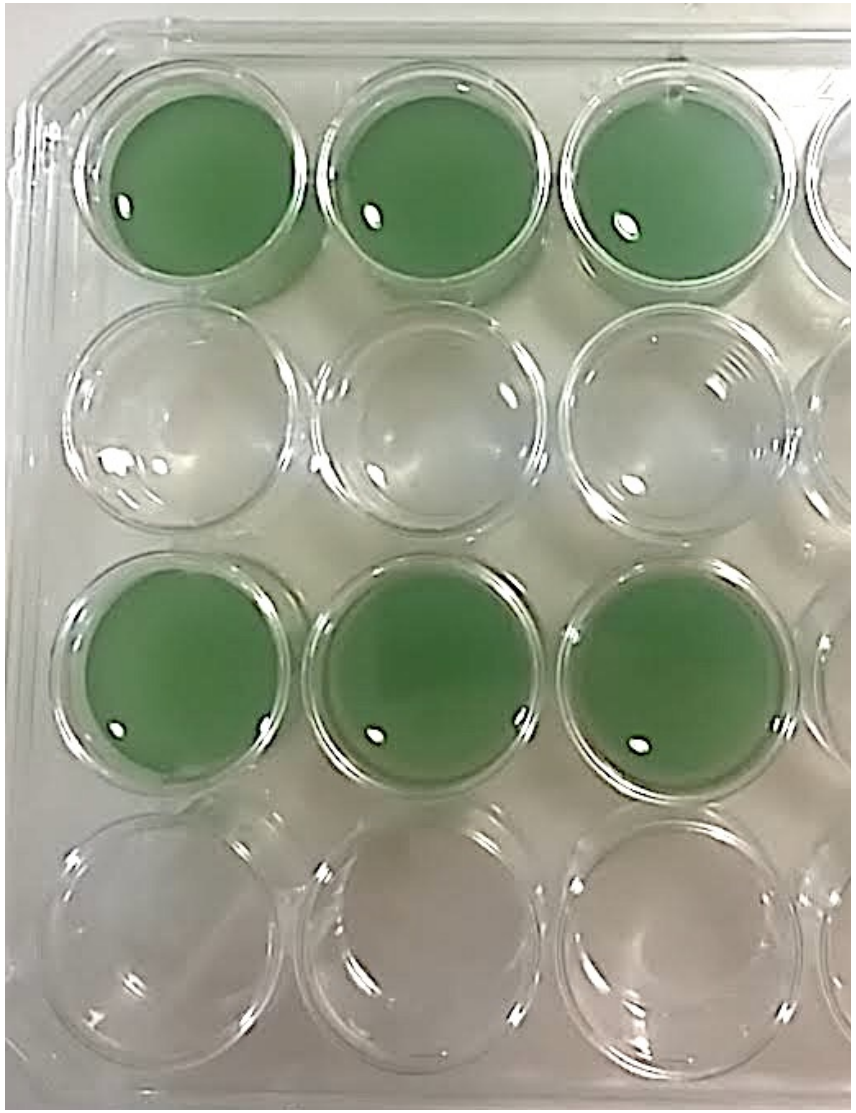

Figure S1. Example of the triplicates of treated and untreated samples (green wells) in 24-wells plates. The plates were kept on a rotary shaker at 37°C for 24 hours. Transparent wells contained 2 mL metal solutions set up as positive control.
