# Supplementary material for: Bioremoval of Yttrium (III), Cerium (III), Europium (III), and Terbium (III) from Single and Quaternary Aqueous Solutions Using the Extremophile Galdieria sulphuraria (Galdieriaceae, Rhodophyta)
Source: Plants (Basel). 2022 May 22;11(10):1376. doi: 10.3390/plants11101376 (PMC9144214; doi:10.3390/plants11101376)
Supplement: Supplementary file 1 [file plants-11-01376-s001.zip › TableS1.pdf]

Table S1. Total metal removed from single and quaternary metal aqueous solutions by *G. sulphuraria*, strain SAG 107.79. Data are expressed as μmol/g dry matter. The total metal removed quantities were calculated by adding the amount of every metal component (Y^{3+} + Ce^{3+} + Eu^{3+} + Tb^{3+}).

		pH 2.5		pH 3.5		pH 4.5		pH 5.5	
	**Metal system**	**Single**	**Quaternary**	**Single**	**Quaternary**	**Single**	**Quaternary**	**Single**	**Quaternary**
**Total metal removed (μmol/g dm)**	**Y3+**	0.39	0.17	0.83	0.23	4.75	1.56	10.44	1.75
	**Ce3+**	0.41	0.15	1.08	0.33	5.94	2.09	10.97	3.11
	**Eu3+**	0.42	0.16	1.44	0.76	10.82	6.21	14.34	6.99
	**Tb3+**	0.40	0.19	1.22	0.62	10.85	5.69	11.13	6.43
	**Total metals**	/	0.66	/	1.93	/	15.56	/	18.28
