# Supplementary material for: Bioremoval of Yttrium (III), Cerium (III), Europium (III), and Terbium (III) from Single and Quaternary Aqueous Solutions Using the Extremophile Galdieria sulphuraria (Galdieriaceae, Rhodophyta)
Source: Plants (Basel). 2022 May 22;11(10):1376. doi: 10.3390/plants11101376 (PMC9144214; doi:10.3390/plants11101376)
Supplement: Supplementary file 1 [file plants-11-01376-s001.zip › TableS2.pdf]

Table S2. Total metal removed from single and quaternary metal aqueous solutions by *G. sulphuraria*, strain ACUF 427. Data are expressed as µmol/g dry matter. The total metal removed quantities were calculated by adding the amount of every metal component ($Y^{3+} + Ce^{3+} + Eu^{3+} + Tb^{3+}$).

		pH 2.5		pH 3.5		pH 4.5		pH 5.5	
	**Metal system**	**Single**	**Quaternary**	**Single**	**Quaternary**	**Single**	**Quaternary**	**Single**	**Quaternary**
**Total metal removed (µmol/g dm)**	**Y3+**	22.43	5.08	20.13	3.56	28.36	4.92	25.25	4.58
	**Ce3+**	20.98	5.16	25.08	3.92	29.82	7.64	42.60	6.59
	**Eu3+**	23.49	10.01	24.66	8.47	36.78	15.17	42.91	13.50
	**Tb3+**	22.26	11.47	24.14	9.02	40.58	15.20	34.24	13.74
	**Total metals**	/	31.72	/	24.96	/	42.93	/	38.41
